# Supplementary material for: Involvement of the p38 MAPK-NLRC4-Caspase-1 Pathway in Ionizing Radiation-Enhanced Macrophage IL-1β Production
Source: Int J Mol Sci. 2022 Nov 9;23(22):13757. doi: 10.3390/ijms232213757 (PMC9698243; doi:10.3390/ijms232213757)

Supplementary Table S1. Primers used for real-time PCR analysis in this study.

Gene	Primer Sequence	T_m (°C)
IL-1β	F: 5’-TGT GTA ATG AAA GAC GGC ACA-3’<br>R: 5’-GGG GAA CTC TGC AGA CTC AA-3’	55.7
NLRC4	F: 5’- TGC CAA ACT TGG ATT GAA AA-3’<br>R: 5’- ATC CGT CAC TGC TCA CAC AG-3’	55
Caspase-1	F: 5’- GCT TGA AAG ACA AGC CCA AG-3’<br>R: 5’- GGC CTT CTT AAT GCC ATC AT-3’	53.7
GAPDH	F: 5’- CAC TCA CGG CAA ATT CAA CG-3’<br>R: 5’- GAC TCC ACG ACA TAC TCA GC-3’	55

Supplementary Table S2. The catalog number, company, and dilution of all antibodies used in this study.

Antibodies for western blotting

Antibody	Company	Catalog No.	Dilution rate
IL-1β	Santa Cruz Biotechnology	Sc-7884	1:1000
NLRC4	Abcam	ab201792	1:1000
NLRP3	R&D systems	MAB7578	1:1000
AIM2	Cell Signaling Technology	#13095	1:1000
Caspase-1	Abcam	ab179515	1:1000
p-p38 MAPK	Cell Signaling Technology	#9215	1:1000
p38 MAPK	Cell Signaling Technology	#9212	1:1000
p-ERK	Cell Signaling Technology	#4370	1:1000
ERK	Cell Signaling Technology	#9102	1:1000
p-JNK	Cell Signaling Technology	#9255	1:1000
JNK	Cell Signaling Technology	#9152	1:1000
Heme Oxygenase-1	Santa Cruz Biotechnology	Sc-10789	1:1000
ACTIN	Sigma-Aldrich	A5441	1:1000

Supplementary Table S2. The catalog number, company, and dilution of all antibodies used in this study. (Continued)

Antibodies for western blotting

Antibody	Company	Catalog No.	Dilution rate
Anti-Rabbit	Invitrogen	G-21234	1:5000
Anti-Mouse	Invitrogen	G-21040	1:5000
Anti-Rat	Invitrogen	G-31470	1:5000

Supplementary Table S2. The catalog number, company, and dilution of all antibodies used in this study. (Continued)

Antibodies for immunofluorecenece

Antibody	Company	Catalog No.	Dilution rate
IL-1β	Santa Cruz Biotechnology	Sc-7884	1:100
NLRC4	ABclonal	A7382	1:100
Caspase-1	Santa Cruz Biotechnology	Sc-514	1:100
DAPI	Vector Laboratories	H-1200	1:100
FITC	Vector Laboratories	FI-1200	1:200
Texas Red	Vector Laboratories	FI-1200	1:200

Supplementary Figure S1. In J774A.1 macrophage cells, ionizing radiation induces increased IL-1β production for LPS. J774A.1 (2×10^5 cells/mL) cells were irradiated (5 Gy) using a blood gamma irradiator. After 24 h, the cells were treated with LPS (0.1 μg/mL). After another 24 h, the supernatant was used for IL-1β (**A**) determination. (**B**,**C**) Representative confocal microscopy image for intracellular IL-1β detection of LPS only- and IR + LPS-treated macrophages and fluorescence analysis data.

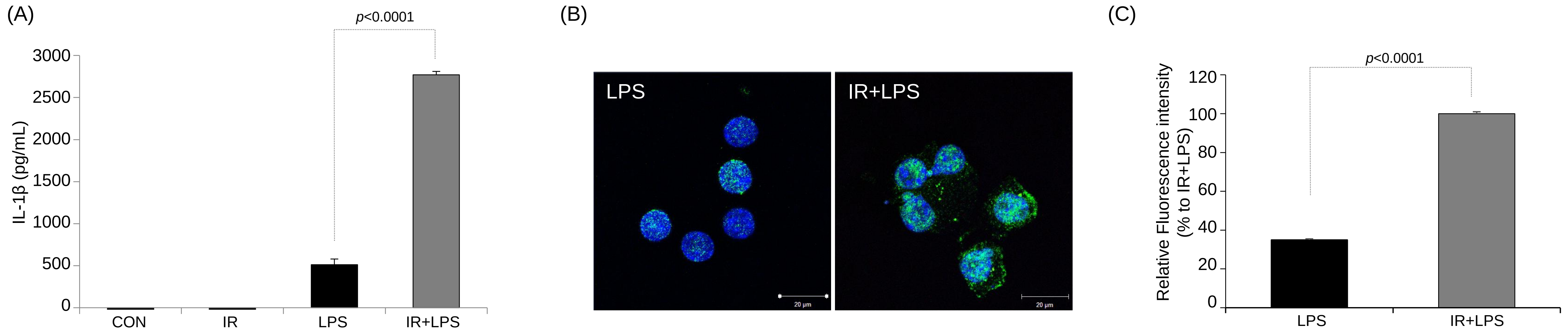

Supplementary Figure S2.

(**A**) Ionizing radiation increased the expression of caspase-1 protein and the cleavage of caspase-1 protein in RAW264.7 macrophage cells. (**B**) The knockdown efficiency of caspase-1 using shRNA was verified using a caspase-1 activity assay kit. (**C**) IR also increased the expression of caspase-1 protein in J774A.1 macrophage cell. (**D**) In J774A.1 cells, when caspase-1 was knocked down using shRNA, the increase in IL-1β production caused by ionizing radiation was suppressed.

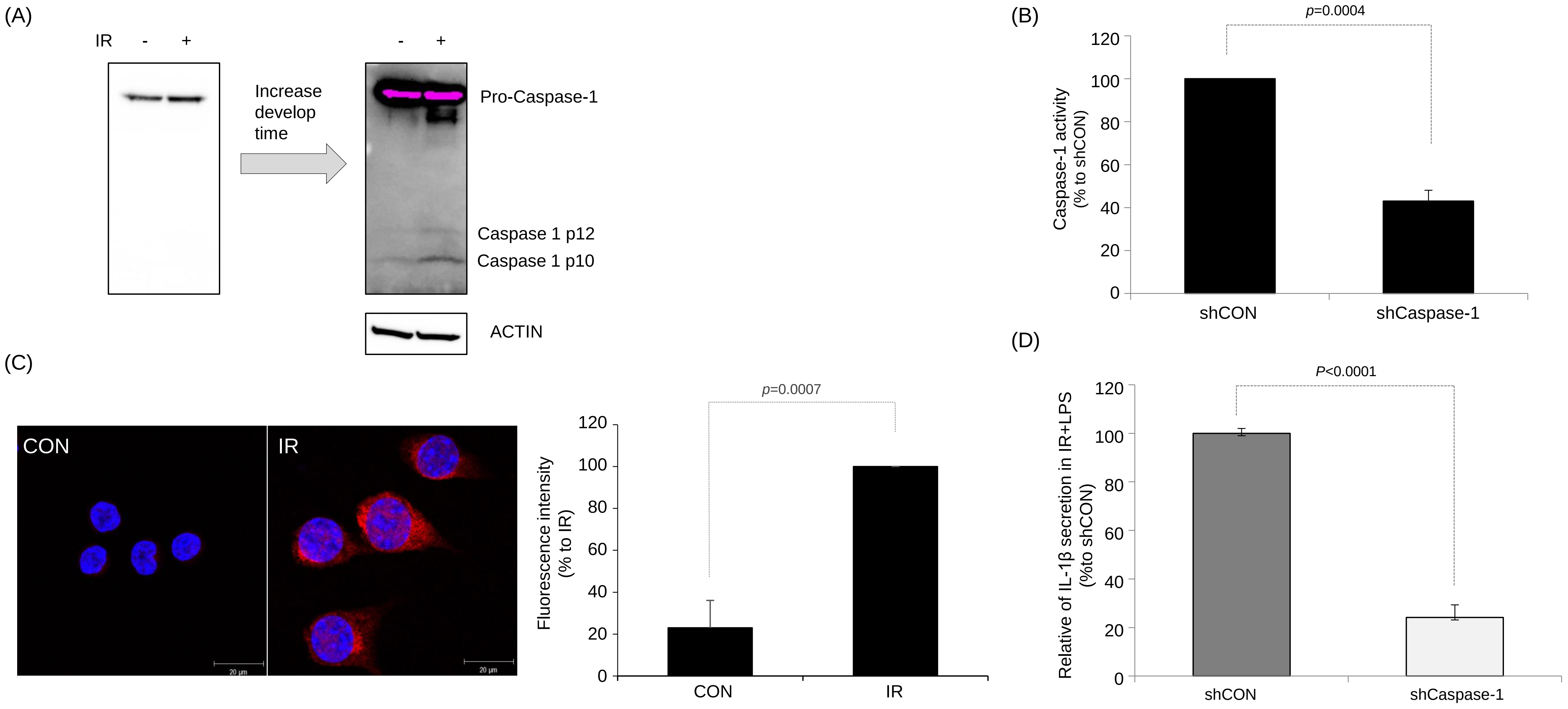

Supplementary Figure S3. In J774A.1 macrophage cells, the expression of caspase-1 protein is increased by IR.

(**A**,**B**) Fluorescence images of RAW264.7 cells after staining with NLRC4; red (PE) indicates NLRC4, and blue (DAPI) indicates the nuclei. Scale bar = 20 μm.

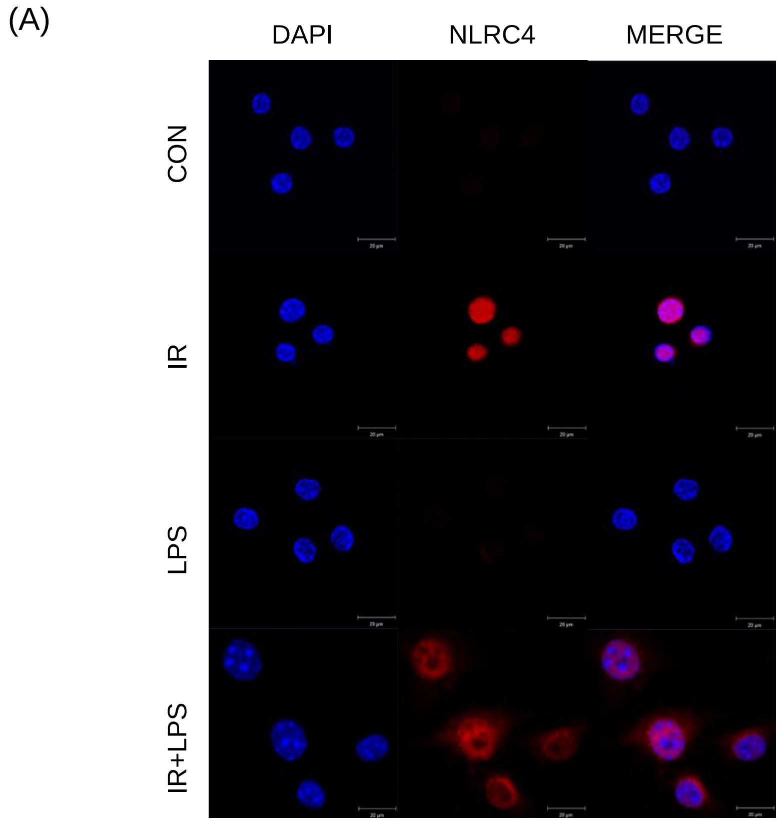

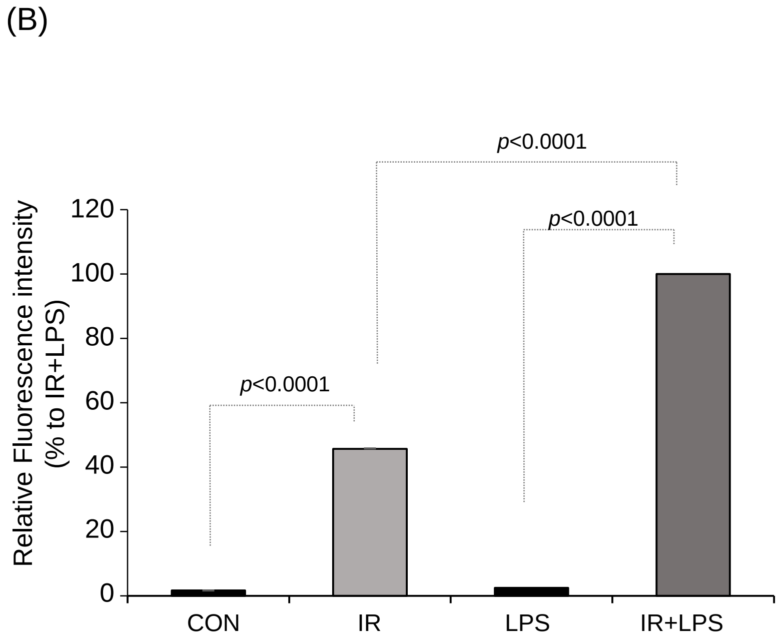

Supplementary Figure S4. In J774A.1 macrophage cells, p38 MAPK is also involved in IR-induced increase in IL-1β production.

(**A**) IR increases the phosphorylation of p38 MAPK in J774A.1 macrophage cells. (**B**) The p38 MAPK inhibitor SB203580 suppresses the IR-enhanced LPS-stimulated IL-1β production in J774A.1 macrophage cells. (**C**) SB203580 did not affect IL-1β production induced by LPS treatment only in RAW264.7 macrophages.

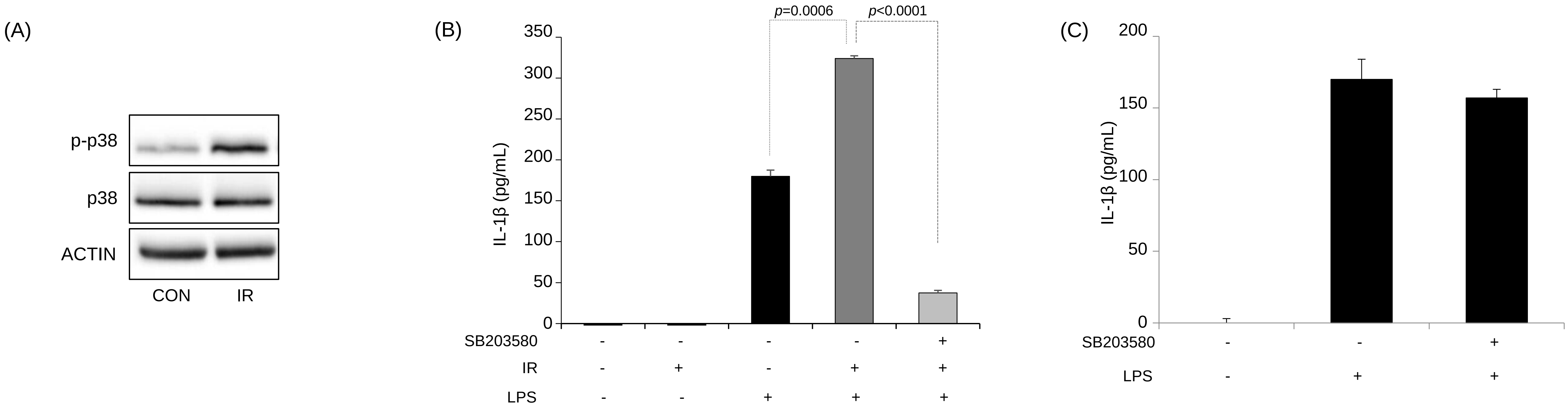

Supplementary Figure S5

(A) Pretreatment with NAC did not inhibit the increase in IL-1β production by irradiation

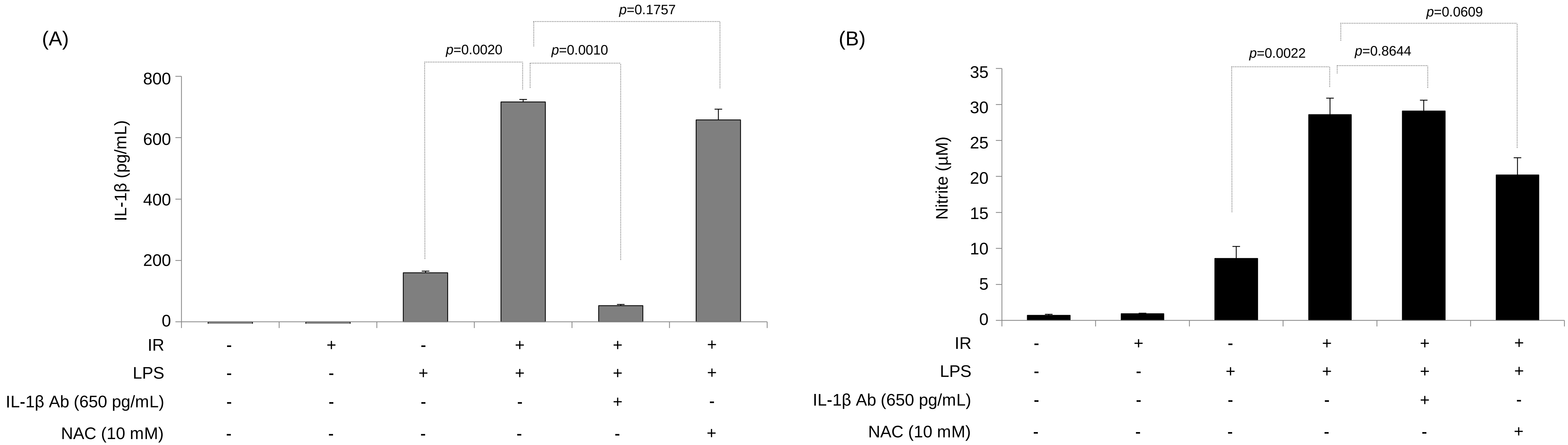

Supplementary Figure S6. None of the three MAPK inhibitors inhibited the increase in NO production by IR + LPS in macrophages

RAW264.7 macrophages were pretreated with ERK (PD98059, 5 µM), JNK (SP600125, 5 µM), and p38 MAPK (SB203580, 5 µM) inhibitors as indicated for 30 min, followed by IR (5 Gy). The cells were treated with LPS (0.1 µg/mL) 24 h later, and NO_2^-(nitrite) was quantified after a further 24 h of incubation.

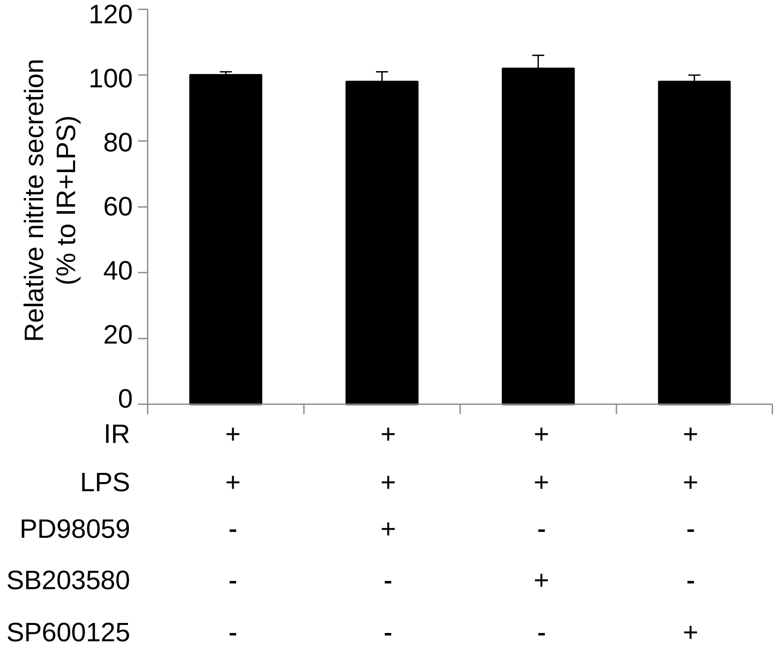

Supplement: Supplementary file 1 [file ijms-23-13757-s001.zip › ijms-1940216-supplementary.pdf]
